# The Relation of ABO Blood Group to the Severity of Coronavirus Disease: A Cross-Sectional Study From a Tertiary Care Hospital in Karachi

**DOI:** 10.7759/cureus.16598

**Published:** 2021-07-23

**Authors:** Fazal U Rehman, Syed Furrukh Omair, Fatima Memon, Bakhtawar J Rind, Danish Ahmed Memon, Syed Ahsan Ali, Bilal Ahmed, Naureen Ali

**Affiliations:** 1 Medicine, The Aga Khan University Hospital, Karachi, PAK; 2 Nephrology, The Aga Khan University Hospital, Karachi, PAK; 3 Internal Medicine, Dr Ruth Pfau Civil Hospital, Karachi, PAK; 4 Family Medicine, The Aga Khan University Hospital, Karachi, PAK; 5 Medicine, Jam Ghulam Qadir Civil Hospital Hub, Quetta, PAK; 6 Acute Medicine, Russells Hall Hospital, Dudley, GBR; 7 Internal Medicine, The Aga Khan University Hospital, Karachi, PAK; 8 School of Nursing and Midwifery Department, The Aga Khan University Hospital, Karachi, PAK

**Keywords:** disease severity, male gender, diabetes, covid-19, abo blood group

## Abstract

Background

Blood groups are considered to have an impact on the occurrence and severity of coronavirus disease. While among Chinese and Caucasian, blood group O individuals were less and group A were more likely to have severe disease and mortality, data on South Asians aren’t available.

Objective

This study aimed to find out the association of disease severity with blood group among coronavirus disease 2019 (COVID-19) patients.

Materials and methodology

Data were collected on a predesigned questionnaire containing details of patient demographics, medical comorbidities, clinical presentation, and laboratory parameters. Multiple logistic regression was used to determine the association of the blood group with the severity of coronavirus disease.

Result

Among the study participants, blood group B has the highest distribution (39.8%), followed by O (30.0), A (21.9%), and AB (8.1%). About three-fourths (69.9%) had mild to moderate disease while 30.0% had severe disease. Age, gender, hypertension, diabetes mellitus, and hemoglobin level were all associated with disease severity among COVID-19 patients in univariate analysis on P-value for selection (<0.25). The final model showed that the odds of disease severity is 3.62 times higher among males (OR: 3.62, 95% CI: 2.15-6.08) and 2.00 times higher among diabetic patients (OR: 2.00, 95% CI: 1.10-3.01) as compared to female and non-diabetic respectively. However, there was no significant association found between blood group and disease severity.

Conclusion

Blood groups don’t have any role in forecasting the severity of coronavirus disease. However, the male gender and diabetics are prone to have severe disease.

## Introduction

On 31st December 2019, 27 cases of pneumonia of unidentified etiology were documented in Wuhan City, Hubei province in China [1].^ ^The causative agent was recognized from throat swab samples conducted by the Chinese Centre for Disease Control and Prevention (CCDC) on 7th January 2020 and was later named severe acute respiratory syndrome coronavirus 2 (SARS-CoV-2). The disease was titled COVID-19 by the World Health Organization (WHO) [2]. The novel coronavirus SARS-CoV-2, causing the novel coronavirus disease-2019 (COVID-19), is currently spreading briskly worldwide; it has been recently declared as a pandemic by WHO. Recent clinical observation advocates that patient age, male sex, and certain chronic medical conditions (e.g., cardiovascular disease, diabetes, chronic obstructive pulmonary disease) appear to represent a risk for the infection of SARS-Cov-2 and higher disease severity [3].

Evidence shows that blood groups have an impact on the occurrence and severity of coronavirus disease 2019 (COVID-19). The vulnerability to viral infections has been previously found to be related to the ABO blood group. For example, the Norwalk virus and Hepatitis B have clear blood group vulnerability [4,5]. It was also reported that blood group O individuals were less likely to become infected by SARS coronavirus [6]. Increased disease severity and high mortality are due to increased cardiovascular events in patients with type A blood group and the fewer cardiovascular events in the type O blood group [7].

There is no literature relating blood groups with the predisposition and severity of COVID-19 from this part of the world. Hence, this study is intended to study the relationship between the ABO blood types and COVID-19 disease severity.

## Materials and methods

The study was designed based on the strobe guidelines. We conducted a retrospective study in tertiary care hospital Karachi, data were collected from the in-patient’s medical record after obtaining permission from the institutional ethical review committee of Aga Khan University Hospital (ERC#2020-5277-11810). All adult patients (aged >18 years) diagnosed with COVID-19 using a nasopharyngeal swab run by PCR technique, with a known blood group, were included in the study. While, COVID-19 cases with super-imposed bacterial infections elicited by high pro-calcitonin levels, positive blood cultures, or labeled as having bacterial infection by a consultant physician were excluded from the study. No patient identifiers were recorded to protect patient’s privacy. A structured questionnaire was designed to collect information regarding patient demographics, comorbid conditions, clinical presentation, disease severity, and laboratory parameter. Disease severity was characterized as per World Health Organization into non-severe, severe, and critical disease [8].

Trained research staff collected the data based on the inclusion criteria and completed all patients’ information on pre-designed performa. A total of 369 patients of COVID-19 with known blood groups were included within the period of three months (1st April-30th June 2020).

## Results

Our study included 369 patients of COVID-19, of which 57.4% (n=212) were male and remaining 42.5% (n = 157) were females. The mean (± SD) age in years of the study participant was 52.8 ± 17.7. Almost 30.0% of patients have greater disease severity while 69.9% have a non-severe disease. Blood group B has the highest distribution (39.8%) among COVID-19 infected patients followed by O (30.0), A (21.9%), and AB (8.1%). Besides, the prevalence of other comorbidities, documenting hypertension as 38.7%, diabetes mellitus as 36.8%, ischemic heart disease as 15.1%, lung disease as 5.6%, and chronic kidney disease as 10.0% (Table 1). 

**Table 1:** Basic characteristics of study participants (N = 369).

Characteristics	n (%)
Age (years), mean ± SD	52.8 ± 17.7
Gender	
Males	212 (57.4)
Females	157 (42.5)
Blood group	
A	81 (21.9)
B	147 (39.8)
O	111 (30.0)
AB	30 (8.1)
Hypertension (HTN)	
No	226 (61.2)
Yes	143 (38.7)
Diabetes mellitus (DM)	
No	233 (63.1)
Yes	136 (36.8)
Ischemic heart disease (IHD)	
No	313 (84.8)
Yes	56 (15.1)
Lung disease	
No	348 (94.3)
Yes	21 (5.6)
Chronic kidney disease (CKD)	
No	332 (89.9)
Yes	37 (10.0)
Disease severity	
No	258 (69.9)
Yes	111 (30.0)
Hemoglobin level (Hb), mean ± SD	11.7 ± 2.6

All independent variables were consid­ered for their potential association with disease severity in univariate analysis using logistic regression for which unadjusted Odds Ratios were calculated. Age, gender, hypertension, diabetes mellitus, and hemoglobin level, were all associated with disease severity among COVID-19 patients in univariate analysis on P-value for selection (< 0.05) as mentioned in Table 2 

**Table 2:** Univariate analysis of factors associated with disease severity among COVID-19 patients of Karachi, Pakistan (N = 369). OR, odds ratio; CI, confidence interval. *P-value of ≤0.05 was considered statistically significant.

Characteristics	OR (95% CI)	P-value
Age (years)	1.04 (1.02-1.05)	<0.001*
Gender		
Males	3.85 (2.30-6.44)	<0.001*
Females	-	
Blood group		
A	1.11 (0.60-2.07)	
B	0.88 (0.51-1.52)	0.55
O	1.57 (0.68-3.63)	
AB	-	
Hypertension (HTN)		
No	-	
Yes	1.61 (1.02-2.54)	0.037*
Diabetes mellitus (DM)		
No	-	
Yes	1.91 (1.22-3.03)	0.004*
Ischemic heart disease (IHD)		
No	-	
Yes	1.94 (1.08-3.49)	0.027
Lung disease		
No	-	
Yes	1.46 (0.58-3.63)	0.419
Chronic kidney disease (CKD)		
No	-	
Yes	0.72 (0.33-1.59)	0.412
Hemoglobin level (Hb)	1.15 (1.05-1.27)	0.001*

The findings from multiple logistic re­gression models are given in Table 3, examining the association between disease severity and the blood group. The final model showed that the odds of disease severity among male patients are 3.62 times greater than female patients (OR: 3.62, 95% CI: 2.15-6.08). Moreover, the odds of disease severity are 2.00 times higher among diabetic patients than non-diabetic patients (OR: 2.00, 95% CI: 1.10-3.01). However, there was no significant association found between blood group and disease severity among COVID-19 patients. 

**Table 3:** Multivariable analysis of factors associated with disease severity among COVID-19 patients of Karachi, Pakistan (N = 369). *P-value of ≤0.05 was considered statistically significant.

Characteristics	OR (95% CI)	P-value
Gender		
Males	3.62 (2.15-6.08)	<0.001*
Females	-	
Blood group		
A	1.18 (0.61-2.26)	0.611
B	0.86 (0.48-1.53)	0.615
O	-	-
AB	1.48(0.61-3.58)	0.380
Diabetes mellitus (DM)		
No	-	
Yes	2.00 (1.10-3.01)	0.018*

## Discussion

In this observational study, we found that COVID-19 disease severity is not associated with any of the ABO blood group types, and the same observations were also found in other studies [9,10]. However, in several studies, it has been found that people with blood A suffer more severe disease as compared to the individual with blood group O [11,12]. The proposed mechanism for higher disease severity in the individual with blood group A includes its positive association with angiotensin-converting enzyme activity and higher expression of adhesion molecules which in turn increase inflammation and hinders circulation [11].

Zhao et al. found that ABO blood groups play role in predisposing the people with blood group A to acquire the disease as opposed to the individual with blood group O [13], but in our study, the blood group B people were predominant that is 39.8% while people from group A were 21% only. The possible reason could be that we included only those patients whose blood groups were already known for some other reasons like blood donation and the different ethnicity of our study population as it does have a role in ABO blood typing [14]. Moreover, almost one-third of our study participants were having a non-severe disease (69.9%) while only 21.9% had severe disease.

Male gender was strongly correlated with disease severity in our study population, congruent to already established evidence [15]. The factors responsible for less severe disease in females include their lower proneness to disease as they are equipped with two copies of the X chromosome which bears the genes involved in the regulation of innate immunity. They also have higher expression toll-like receptors-7 (TLR7) and CD4+ lymphocytes which play a key role in the defense against viral infection and the clearance of viruses respectively [16].

We also found that diabetic patients were having more severe COVID-19 disease as compared to their counterparts [17]; possible reasons include the metabolic changes in diabetic patients, chronic inflammation, attenuation of both innate and adaptive immune responses predisposing to secondary infection [18]. Moreover, diabetic patients have a higher expression of angiotensin-converting enzyme 2, which facilitates viral uptake thereby increase chances of having severe disease than non-diabetic patients [19,20].

The findings of our study are important; however, it was a small observation single-center study, so the findings can't be generalized. Studies conducted in multiple centers on diverse populations will help in establishing the relationship of ABO blood groups with COVID-19 disease severity 

## Conclusions

 We found no association between blood groups and COVID-19 disease severity. However, male gender and diabetes mellitus were strongly associated with disease severity, warranting more vigilant care.

## References

[1] Lu H, Stratton CW, Tang YW (2020). Outbreak of pneumonia of unknown etiology in Wuhan, China: the mystery and the miracle. J Med Virol.

[2] Singh TK, Kumar S, Tiwari P (2021). Singh TK, Kumar S, Tiwari P. Review on COVID-19 (coronavirus disease). https://www.pharmatutor.org/articles/review-on-covid-19-corona-virus-disease.

[3] Chen N, Zhou M, Dong X (2020). Epidemiological and clinical characteristics of 99 cases of 2019 novel coronavirus pneumonia in Wuhan, China: a descriptive study. Lancet.

[4] Batool Z, Durrani SH, Tariq S (2017). Association of ABO and Rh blood group types to hepatitis B, hepatitis C, HIV and syphilis infection, a five year’ experience in healthy blood donors in a tertiary care hospital. J Ayub Med Coll Abbottabad.

[5] Lindesmith L, Moe C, Marionneau S (2003). Human susceptibility and resistance to Norwalk virus infection. Nat Med.

[6] Cheng Y, Cheng G, Chui CH (2005). ABO blood group and susceptibility to severe acute respiratory syndrome. JAMA.

[7] Wu O, Bayoumi N, Vickers MA, Clark P (2008). ABO(H) blood groups and vascular disease: a systematic review and meta-analysis. J Thromb Haemost.

[8] Maguire BJ, Guérin PJ (2020). A living systematic review protocol for COVID-19 clinical trial registrations. Wellcome Open Res.

[9] Latz CA, DeCarlo C, Boitano L (2020). Blood type and outcomes in patients with COVID-19. Ann Hematol.

[10] Liumbruno GM, Franchini M (2013). Beyond immunohaematology: the role of the ABO blood group in human diseases. Blood Transfus.

[11] Dai X (2020). ABO blood group predisposes to COVID-19 severity and cardiovascular diseases. Eur J Prev Cardiol.

[12] Hoiland RL, Fergusson NA, Mitra AR (2020). The association of ABO blood group with indices of disease severity and multiorgan dysfunction in COVID-19. Blood Adv.

[13] Fan Q, Zhang W, Li B, Li DJ, Zhang J, Zhao F (2020). Association Between ABO Blood Group System and COVID-19 Susceptibility in Wuhan. Front Cell Infect Microbiol.

[14] Garratty G, Glynn SA, McEntire R (2004). ABO and Rh(D) phenotype frequencies of different racial/ethnic groups in the United States. Transfusion.

[15] Jin JM, Bai P, He W (2020). Gender differences in patients with COVID-19: focus on severity and mortality. Front Public Health.

[16] Conti P, Younes A (2020). Coronavirus COV-19/SARS-CoV-2 affects women less than men: clinical response to viral infection. J Biol Regul Homeost Agents.

[17] Kumar A, Arora A, Sharma P (2020). Is diabetes mellitus associated with mortality and severity of COVID-19? A meta-analysis. Diabetes Metab Syndr.

[18] Toniolo A, Cassani G, Puggioni A, Rossi A, Colombo A, Onodera T, Ferrannini E (2019). The diabetes pandemic and associated infections: suggestions for clinical microbiology. Rev Med Microbiol.

[19] Lu R, Zhao X, Li J (2020). Genomic characterisation and epidemiology of 2019 novel coronavirus: implications for virus origins and receptor binding. Lancet.

[20] Zhang H, Penninger JM, Li Y, Zhong N, Slutsky AS (2020). Angiotensin-converting enzyme 2 (ACE2) as a SARS-CoV-2 receptor: molecular mechanisms and potential therapeutic target. Intensive Care Med.

